# Oxytocin administration suppresses hypothalamic activation in response to visual food cues

**DOI:** 10.1038/s41598-017-04600-0

**Published:** 2017-06-27

**Authors:** Agatha A. van der Klaauw, Hisham Ziauddeen, Julia M. Keogh, Elana Henning, Sekesai Dachi, Paul C. Fletcher, I. Sadaf Farooqi

**Affiliations:** 1 University of Cambridge Metabolic Research Laboratories and the NIHR Cambridge Biomedical Research Centre, Wellcome Trust-MRC Institute of Metabolic Science, Addenbrooke’s Hospital, Cambridge, UK; 2 Department of Psychiatry, University of Cambridge, Cambridge Biomedical Campus, Cambridge, UK; 3 Cambridgeshire and Peterborough NHS Foundation Trust, Cambridge, UK

## Abstract

The aim of this study was to use functional neuroimaging to investigate whether oxytocin modulates the neural response to visual food cues in brain regions involved in the control of food intake. Twenty-four normal weight volunteers received intranasal oxytocin (24 IU) or placebo in a double-blind, randomized crossover study. Measurements were made forty-five minutes after dosing. On two occasions, functional MRI (fMRI) scans were performed in the fasted state; the blood oxygen level-dependent (BOLD) response to images of high-calorie foods versus low-calorie foods was measured. Given its critical role in eating behaviour, the primary region of interest was the hypothalamus. Secondary analyses examined the parabrachial nuclei and other brain regions involved in food intake and food reward. Intranasal oxytocin administration suppressed hypothalamic activation to images of high-calorie compared to low-calorie food (P = 0.0125). There was also a trend towards suppression of activation in the parabrachial nucleus (P = 0.0683). No effects of intranasal oxytocin were seen in reward circuits or on *ad libitum* food intake. Further characterization of the effects of oxytocin on neural circuits in the hypothalamus is needed to establish the utility of targeting oxytocin signalling in obesity.

## Introduction

Obesity is associated with significant complications including type 2 diabetes, cardiovascular disease and some forms of cancer. However, effective pharmacological therapies for weight loss remain limited. There has been recent interest in the peptide oxytocin which appears to play a role in energy homeostasis, in addition to its role in parturition and lactation. Centrally administered oxytocin decreases food intake in a dose-dependent manner in lean and obese rodents^1^. Additionally, targeted deletion of the oxytocin or oxytocin receptor gene in mice results in late onset obesity^2, 3^. In humans, loss of function variants of oxytocin or its receptor have not been described to date, but a 42% reduction in oxytocin neurons in the hypothalamus has been found in post-mortem brain studies of patients with Prader-Willi Syndrome, a genetic syndrome characterised by severe hyperphagia and obesity^4^. Similarly, haplo-insufficiency for SIM1, a transcription factor that is critical for the development of hypothalamic oxytocin neurons, is characterised by severe obesity and hyperphagia in humans^5^ and mice^6^.

Whilst there is compelling evidence for oxytocin’s role in food intake and energy homeostasis in preclinical models, the three human studies to date that have examined the effects of intranasal oxytocin on food intake in humans have shown varying results^7–10^. It therefore remains to be determined whether intranasal oxytocin acutely modulates the neural circuitry involved in the regulation of food intake in humans. We examined the effect of a single dose of intranasal oxytocin, compared to placebo, on the neural response to visual food cues using functional magnetic resonance imaging (fMRI) in healthy lean volunteers. We used an experimental paradigm that has been used by ourselves and others^11^, to investigate the neural response to visual food cues, and its modulation by physiological and pharmacological manipulations^12, 13^. We also examined the effects of intranasal oxytocin on food intake.

## Methods

### Participants

Twenty-four healthy volunteers (n = 11 male; mean ± SD, BMI 22.3 ± 0.4 kg/m² (range 17–25 kg/m^2^); age 27.1 ± 1.6 yrs (range 21–59 yrs)) were enrolled in the study after providing written informed consent. The study was approved by the Cambridge South Research Ethics Committee (LREC 12/EE/0091), UK and conducted in accordance with the Declaration of Helsinki. Participants were required to be weight stable for the 3 months prior to the study. Exclusion criteria were significant medical history, pregnancy, breastfeeding, use of any regular medication and any contraindications to MRI scanning. If participants were suffering from hay fever or common cold, study visits were rescheduled as nasal inflammation may impair absorption of oxytocin.

### Design

In a double-blind, randomized crossover four-period design, participants received placebo (2 treatment periods) or 24 IU oxytocin (Syntocinon, Novartis, 3 sprays per nostril, 2 treatment periods). Participants attended for a total of 4 visits, separated by at least one week to avoid carry-over effects. Participants were instructed to fast overnight prior to each visit and received either placebo or oxytocin at the beginning of each study visit. Following this, 45 minutes later they underwent fMRI scanning on 2 visits and received an ad libitum breakfast on 2 visits.

### Procedures

#### Imaging paradigm

Participants performed a simple task adapted from one reported previously^11^ in which they viewed and reported their subjective liking for images from four categories: high-calorie foods (e.g. pizza), low-calorie foods (e.g. carrots), rewarding non-food items (e.g. consumer gadgets) and less rewarding non-food items (e.g. paper clips). All images were matched for colour, size and background. The task comprised of 6 blocks of 5 images each for each category, randomly interspersed with fixation blocks. A total of 120 images were presented, each image for 4.5 seconds (block length = 22.5 seconds). Participants were instructed to indicate how much they liked each image by pressing a button on the button box and were instructed that the duration of their button press would be taken as the measure of their liking.

### Data Acquisition and Processing

fMRI data were acquired on a Siemens Verio scanner operating at 3 Tesla with a 192 mm field of view at the Wolfson Brain Imaging Centre, Cambridge, UK. 446 gradient echo T2*-weighted echo planar images (EPI) were acquired for each participant. To avoid T1 equilibration effects, the first six images were discarded. The images comprised 31 slices, each 3 mm thick with a 0.8 mm inter-slice gap and a 64 × 64 data matrix. Slices were acquired in an ascending interleaved fashion with a repetition time = 2000 ms, an echo time = 30 ms, flip angle = 78°, axial orientation = oblique. Data were analysed using the SPM8 program (www.fil.ion.ucl.ac.uk) for statistical parametric mapping. Images were realigned to the mean image and then spatially normalised to a standard template. As the hypothalamus was the defined region of interest (ROI) the data were spatially smoothed with an isotropic 4 mm full width at half maximum 3 dimensional Gaussian kernel. The time series in each session were high-pass filtered (with cut-off frequency 1/120 Hz) and serial autocorrelations were estimated using an AR (1) model.

### Imaging Analysis

The four experimental categories were modelled using a box car function convolved with a canonical haemodynamic response. The first level models included temporal derivatives for each condition, a regressor that contained the mean button press duration for the block, and the motion realignment parameters. The beta parameter estimated at each voxel in the general linear model for each stimulus type was derived from the mean least-squares fit of the model to the data. The contrast of interest was the comparison of high-calorie to low-calorie foods. The primary region of interest (ROI) was the hypothalamus and the following exploratory ROIs were also examined: the parabrachial nuclei, ventral striatum, amygdala, caudate and putamen bilaterally, and the midbrain. The hypothalamus, amygdala, caudate and putamen ROIs were generated using the PickAtlas tool in SPM8. As the hypothalamus ROI is small, we overlaid the hypothalamus ROI on the mean structural image from all 24 subjects (left) panel and on the SPM template mean structural image and found that the ROI defined in PickAtlas fitted the data. The parabrachial nuclei were defined as 5 mm spheres centred on −8, −36, −26 (left PBN) and 6, −36, −22 (right PBN)^14^. The ventral striatum and midbrain masks were defined based on previously published anatomical masks^15^. In case of bilateral ROIs we did not pool the left and right ROIs. The average parameter estimate for all voxels within the mask were extracted from all ROIs separately using MARSBAR. After unblinding the data, 17 participants had received placebo, and 7 had received oxytocin at their first visit. Therefore a visit factor was included in all analysis models. The extracted parameter estimates were examined using a mixed-effect model in R (nlme) with subject as a random effect, and treatment condition and visit as fixed effects. In addition to the ROI analysis, an exploratory whole brain analysis was carried out at a statistical threshold of p < 0.05, family-wise error corrected.

### Food intake study

An ad libitum breakfast meal paradigm was used to measure food intake. Two nutritionally balanced (30% fat, 20% protein and 50% carbohydrate energy content) foods familiar to British people were provided in excess (14.4 MJ/3352 Kcal). Volunteers were instructed to eat until comfortably full and food was covertly weighed before and after consumption. Participants were given various computer tasks and questionnaires between dosing and food intake and were instructed that these were the primary outcome measures to avoid awareness of monitoring food intake as this might have influenced their behaviour. Food intake was examined using mixed effect models in R; p < 0.05 was deemed to be statistically significant. Visit order was included in all models.

## Results

**Figure 1:**
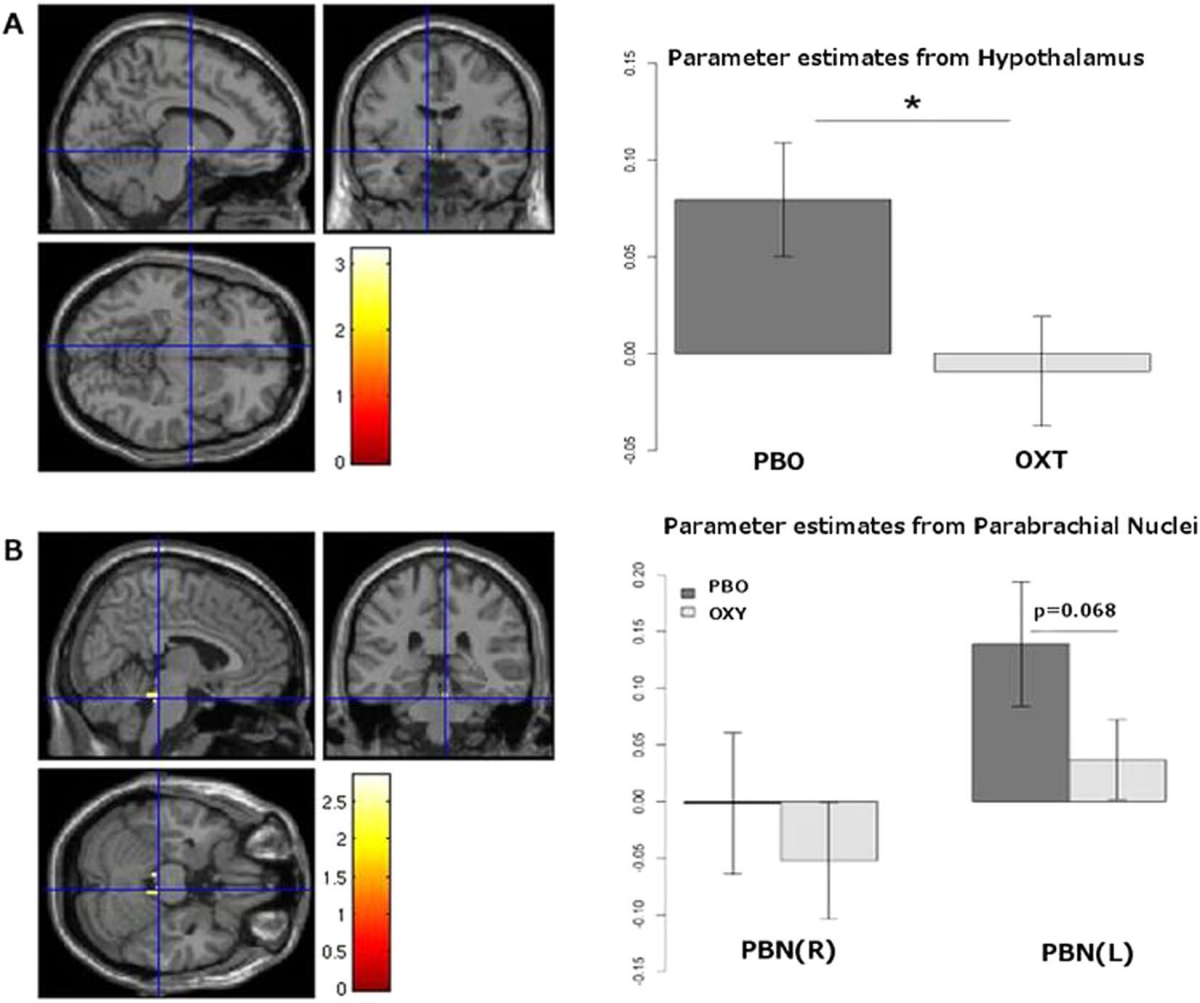
Hypothalamic and parabrachial nuclei (PBN) response to oxytocin. **A: (A)** Examination of the contrast of high calorie versus low calorie foods within the hypothalamus across all datasets (N = 48). The statistical parametric map is overlaid on the SPM8 T1 template image and thresholded at p < 0.05 uncorrected for display purposes. After small volume correction, one voxel −8, −6, −8 survives at p = 0.0125. **(B)** Parameter estimates extracted from the hypothalamus ROIs showing a significant decrease in the parameter estimate in the oxytocin (OXT) condition compared to placebo (PBO). **B: (A)** Examination of the contrast of high calorie versus low calorie foods within the PBN across all datasets (N = 48). Image is thresholded at p < 0.05 uncorrected for display purposes. **(B)** Parameter estimates extracted from the PBN ROIs showing a trend towards decrease in the parameter estimate in the right PBN in the oxytocin condition (p = 0.0683).

All 24 participants completed the both neuroimaging visits and 23 completed both food intake visits, one participant only attended one food intake visit. In the primary ROI analysis of the hypothalamus, the BOLD response to high-calorie vs low-calorie food images, was significantly attenuated with oxytocin compared to placebo (*T* = −2.72, *df* = 22, p = 0.0125, Fig. 1). In addition, we found a trend towards a suppressive effect of oxytocin in the right parabrachial nucleus (PBN) (*T* = −1.92, *df* = 22, p = 0.0683, Fig. 1). There were no effects of oxytocin seen in the ventral striatum, amygdala, caudate, putamen or midbrain (Supplementary Figure 1). No other regional differences were found in the exploratory whole brain analysis. We explored the food vs non-food contrast in the primary and exploratory ROIs; these effects were not significant (Supplementary Figure 2). There were no differences between males and females (data not shown).

**Figure 2:**
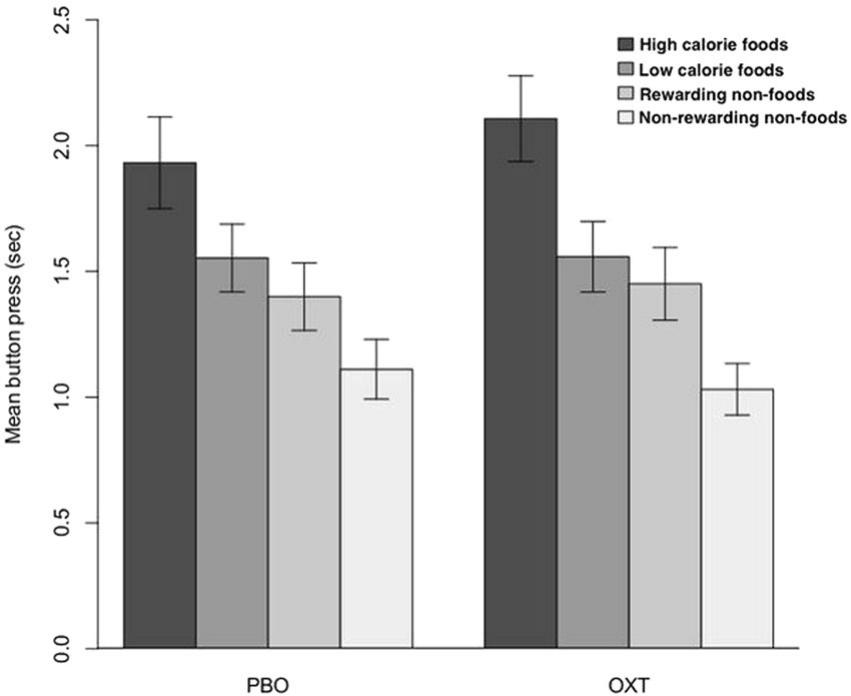
Liking ratings for visual food cues. Subjective liking ratings for different food cues by treatment condition (PBO-placebo, OXT-oxytocin).

Although we observed that liking ratings were greater for high calorie rather than low calorie food images (Fig. 2), there was no difference in subjective liking measures for any of the image categories between the placebo and oxytocin conditions (Fig. 2). Additionally, energy intake at an ad libitum test meal was comparable in the placebo (745 ± 73 kcal) and oxytocin (761 ± 53 kcal) treatments. There were no significant correlations between the degree of hypothalamic suppression on oxytocin and food intake with placebo or oxytocin.

## Discussion

Here we show that intranasal oxytocin modulates hypothalamic activation to visual food cues in humans. Neural activation (increase in the BOLD signal) by images of high calorie compared to low calorie foods may be a proxy for the brain response to hunger or for increased salience of more rewarding food stimuli in the hungry state, based on previous studies in states of complete leptin deficiency^11^. We showed that oxytocin administration suppresses BOLD activation by images of high calorie compared to low calorie foods in the hypothalamus. This suggests that oxytocin directly/indirectly modulates neural circuits within the hypothalamus that are normally activated by images of high calorie compared to low calorie foods (ie: by hunger). These findings are intriguing and warrant replication in larger studies of lean and obese people. As changes in hypothalamic activation as visualised by fMRI have been shown to correlate with the anorectic effects of anti-obesity drugs such as sibutramine^13^ and exenatide^16^ these findings suggest that further exploration of therapeutic agents which might engage these neural circuits sufficiently to affect food intake is warranted.

We did not find an effect of oxytocin on *ad libitum* food intake in our paradigm in line with a study in 20 healthy lean men^9^, but in contrast with a recent study of lean and obese men^8^. Potential explanations are the inclusion of lean and obese people in the latter study or the timing of the test meal (45 minutes in the studies that did not show effect on breakfast intake and 60 minutes in the study that did). Indeed, Ott *et al*. did find a decrease in snacking throughout the morning after oxytocin administration^9^. Additionally, one perturbation in the complex neural circuitry controlling eating behaviour (ie: reduced activation of a set of circuits in the hypothalamus) may not be sufficient to generate an anorectic effect that could be detected within the constraints of our food intake test.

The neural circuits that convey the anorectic effects of oxytocin involve areas with high oxytocin receptor expression such as the ventromedial hypothalamus, the ventral tegmental area, the nucleus accumbens and amygdala. Interestingly, a recent study by Striepens *et al*. found that intranasal oxytocin enhanced cognitive control in a food craving task alongside neural activation of prefrontal cortical areas involved in inhibitory control^17^.

There are some limitations with our study design. Having participants return for four visits may have had an impact on their behavior through familiarity with the experimental paradigm. We did include a visit effect in all the models to account for the repeated within-subject testing. This was however done separately for the fMRI and food intake sessions.

Whilst intranasal administration was directly supervised in all cases, there is no straightforward way of measuring uptake of intranasally administered drugs. There are inherent challenges in assessing the central bio-availability of oxytocin following intranasal administration^18^. Although small increases in CSF levels of oxytocin (from 20 to 30 pM) have been measured after intranasal administration in humans^17^, central availability is likely to vary greatly between and within individuals. Indeed, a recent study has suggested that oxytocin might be more effective at suppressing food intake in obese compared to lean participants^10^. The latter findings are supported by rodent studies where weight loss is more pronounced in diet-induced obese (DIO) animals compared to lean controls^19^ following oxytocin administration. Interestingly, a small prospective study with obese men and women found small effects on body weight during 8 week treatment with four times daily intranasal oxytocin^7^. Our human studies suggest that, as identified in the autism field^20^, dose and duration of effect (and thus timing of any physiological measurement) are important issues when considering oxytocin as a potential therapeutic. It is plausible that repeated dosing may increase central bioavailability, but this requires replication.

In conclusion, we have demonstrated that intranasal oxytocin modulates hypothalamic activation to visual food cues in humans. These findings suggest that oxytocin responsive neural circuits contribute to the hypothalamic response to visual food cues. Further characterisation of these neural circuits will be needed to determine whether modulation of oxytocin-mediated signalling may be of benefit in the treatment of disorders of energy homeostasis such as obesity.

## Electronic supplementary material

Supplementary material
