# Supplementary material for: Oxytocin administration suppresses hypothalamic activation in response to visual food cues
Source: Sci Rep. 2017 Jun 27;7:4266. doi: 10.1038/s41598-017-04600-0 (PMC5487325; doi:10.1038/s41598-017-04600-0)

Supplementary Material

Oxytocin administration suppresses hypothalamic activation in response to visual food cues

Agatha A van der Klaauw, Hisham Ziauddeen Julia M. Keogh, Elana Henning, Sekesai Dachi, Paul C. Fletcher, I. Sadaf Farooqi

Supplementary Figure 1: Treatment effects in all ROIs for the contrast of high-calorie versus low-calorie food images

The bars represent the least square means of the parameter estimates from the ROIs, accounting for visit effects, the error bars represent the standard error of the least square means. Significant treatment effects are only seen in the hypothalamus and there is a trend towards statistical significance in the right parabrachial nucleus (AMG-amygdala, CAU-caudate, HYP-hypothalamus, INS-insula, MID-midbrain, PBN-parabrachial nucleus, PUT-putamen, VS-ventral striatum, L-left, R-right, PBO-placebo, OXT-oxytocin).

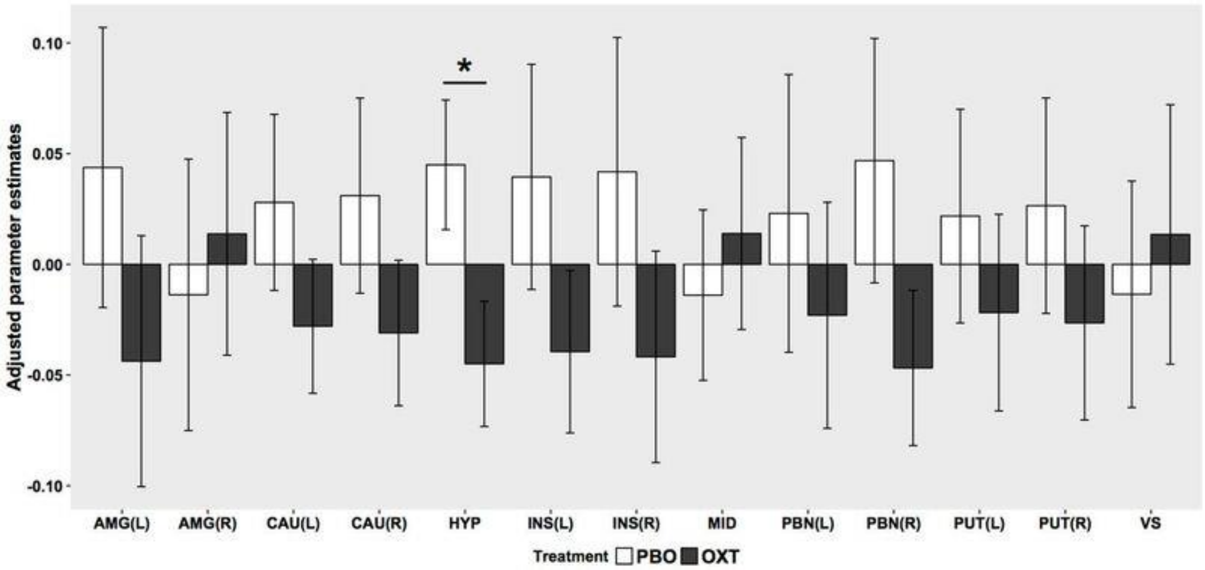

Supplementary Figure 2: Treatment effects in all ROIs for the contrast of food versus non-food images

The bars represent the least square means of the parameter estimates from the ROIs, accounting for visit effects, the error bars represent the standard error of the least square means. No significant treatment effects are seen in any of the ROIs (AMG-amygdala, CAU-caudate, HYP-hypothalamus, INS-insula, MID-midbrain, PUT-putamen, VS-ventral striatum, L-left, R-right, PBO-placebo, OXT-oxytocin).

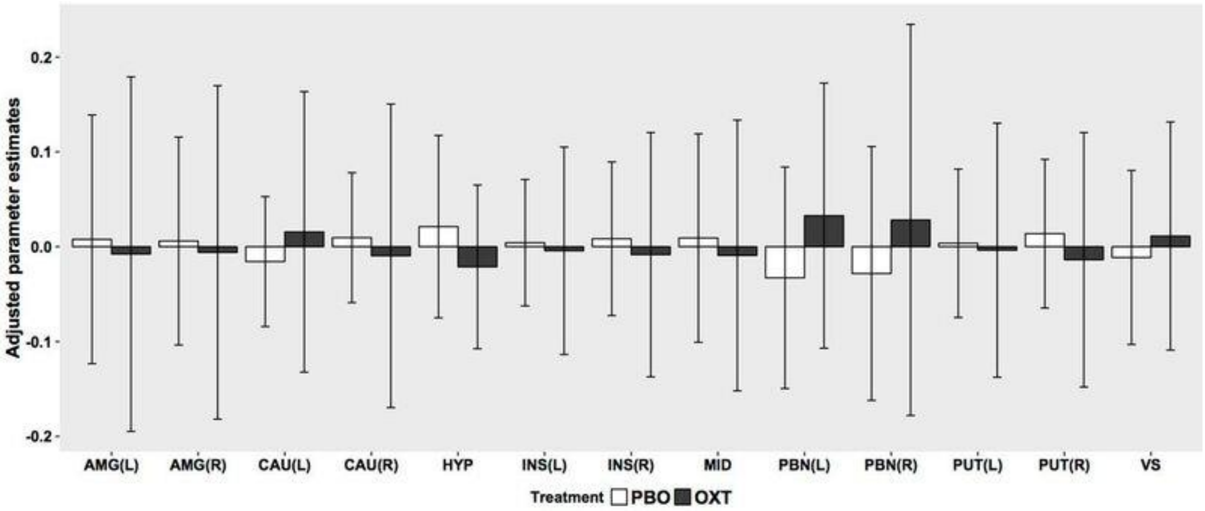

Supplement: Supplementary file 1 — Supplementary material [file 41598_2017_4600_MOESM1_ESM.pdf]
